# Demyelination in Patients with POST-COVID Depression

**DOI:** 10.3390/jcm13164692

**Published:** 2024-08-09

**Authors:** Marina Khodanovich, Mikhail Svetlik, Daria Kamaeva, Anna Usova, Marina Kudabaeva, Tatyana Anan’ina, Irina Vasserlauf, Valentina Pashkevich, Marina Moshkina, Victoria Obukhovskaya, Nadezhda Kataeva, Anastasia Levina, Yana Tumentceva, Svetlana Vasilieva, Evgeny Schastnyy, Anna Naumova

**Affiliations:** 1 Laboratory of Neurobiology, Research Institute of Biology and Biophysics, Tomsk State University, 36 Lenina Ave., Tomsk 634050, Russia; 2 Mental Health Research Institute, Tomsk National Research Medical Center of the Russian Academy of Sciences, Tomsk 634014, Russia; 3 Department of Radiology, School of Medicine, South Lake Union Campus, University of Washington, 850 Republican Street, Seattle, WA 98109, USA; 4 Cancer Research Institute, Tomsk National Research Medical Center of the Russian Academy of Sciences, 12/1 Savinykh Street, Tomsk 634028, Russia; 5 Department of Fundamental Psychology and Behavioral Medicine, Siberian State Medical University, 2 Moskovskiy Trakt, Tomsk 634050, Russia; 6 Department of Neurology and Neurosurgery, Siberian State Medical University, 2 Moskovskiy Trakt, Tomsk 634028, Russia; 7 Medica Diagnostic and Treatment Center, 86 Sovetskaya Street, Tomsk 634510, Russia

**Keywords:** COVID-19, post-COVD, long COVID, depression, demyelination, white matter, magnetic resonance imaging, MRI, macromolecular proton fraction, MPF, major depressive disorder

## Abstract

**Background:** Depression is one of the most severe sequelae of COVID-19, with major depressive disorder often characterized by disruption in white matter (WM) connectivity stemming from changes in brain myelination. This study aimed to quantitatively assess brain myelination in clinically diagnosed post-COVID depression (PCD) using the recently proposed MRI method, macromolecular proton fraction (MPF) mapping. **Methods:** The study involved 63 recovered COVID-19 patients (52 mild, 11 moderate, and 2 severe) at 13.5 ± 10.0 months post-recovery, with matched controls without prior COVID-19 history (n = 19). A post-COVID depression group (PCD, n = 25) was identified based on psychiatric diagnosis, while a comparison group (noPCD, n = 38) included participants with neurological COVID-19 complications, excluding clinical depression. **Results:** Fast MPF mapping revealed extensive demyelination in PCD patients, particularly in juxtacortical WM (predominantly occipital lobe and medial surface), WM tracts (inferior fronto-occipital fasciculus (IFOF), posterior thalamic radiation, external capsule, sagittal stratum, tapetum), and grey matter (GM) structures (hippocampus, putamen, globus pallidus, and amygdala). The noPCD group also displayed notable demyelination, but with less magnitude and propagation. Multiple regression analysis highlighted IFOF demyelination as the primary predictor of Hamilton scores, PCD presence, and severity. The number of post-COVID symptoms was a significant predictor of PCD presence, while the number of acute symptoms was a significant predictor of PCD severity. **Conclusions:** This study, for the first time, reveals extensive demyelination in numerous WM and GM structures in PCD, outlining IFOF demyelination as a key biomarker.

## 1. Introduction

An outbreak of a coronavirus in late 2019 (COVID-19) caused by the severe acute respiratory syndrome coronavirus 2 (SARS-CoV-2) infection quickly became a pandemic. Based on the World Health Organization statistics, there will be 771,820,937 confirmed cases of COVID-19 globally by November 2023, including 6,978,175 deaths (WHO Coronavirus (COVID-19) Dashboard). Many studies indicate the persistence of COVID-19 symptoms following recovery of the acute infection despite clearance of the virus from the body [1,2,3]. This phenomenon of the COVID-19 symptoms persisting for more than 12 weeks following the infection has been characterized as post-COVID-19 syndrome or long-COVID-19 syndrome.

The long-term neurological and psychiatric complications of the COVID-19 infection are being intensively studied. The complications of COVID-19 include “brain fog”, fatigue, headaches, sleep disorders, cognitive impairment, impaired sense of smell and taste, depression, anxiety, sleep disturbances, post-traumatic disorder, and obsessive-compulsive symptoms [4,5,6,7,8,9,10,11,12,13,14].

Among the complications related to COVID-19, depression represents one of the major public health concerns; it decreases quality-of-life outcomes and may cause disability [15]. People who survived the acute phase of COVID-19 have been found to be at increased risk of mental health disorders, including being diagnosed with major depressive disorder (MDD) more often [16]. There are still too few studies to confidently judge whether PCD has any specific features compared to MDD. Some publications described differences in symptoms between PCD and MDD. Simonetti et al. [17] found a high level of excitatory symptoms in post-COVID syndrome, which is more likely to correspond to a mixed form of depression. In contrast, the comparative analysis of biomarkers suggested a similar etiopathogenesis and inflammatory hypothesis for post-COVID depression and MDD [18].

The etiology of MDD is commonly considered to be multifactorial [19]. The pathological mechanisms underlying post-COVID depressive symptoms are mainly related to the inflammation triggered by the peripheral immune-inflammatory response to the viral infection [18,20]. Several studies have also demonstrated neuroinflammation [21,22] in post-COVID syndrome. Neuroinflammation-associated reactivation of microglia and astrocytes is accompanied by the release of cytokines and pro-inflammatory and cytotoxic factors. This change in the extracellular environment negatively affects the functions of myelinating oligodendrocytes and oligodendrocyte precursors [23,24,25]. This leads to the hypothesis that neuroinflammation and subsequent demyelination may be a major cause of post-COVID depression.

Several studies of brain demyelination in post-COVID patients support this hypothesis. White matter damage was seen in 57% of 4342 hospitalized COVID-19 patients [26]. Reports indicated that COVID-19 infection triggers demyelinating diseases such as multiple sclerosis [27], acute disseminated encephalomyelitis [28], and Guillain-Barré syndrome [29,30]. The neurotoxic action of the virus is mediated by binding to angiotensin-converting enzyme-2 (ACE2) receptors or indirectly by inducing a cytokine storm leading to disruption of the blood–brain barrier, immunological mediation, increasing blood coagulation, and acting as a trigger for autoimmune-mediated demyelinating injuries in the CNS [31,32]. Recent MRI studies have shown widespread alterations of white matter (WM) microstructure in the brain after COVID-19, and an increase in radial diffusivity is indicative of demyelination and axonal damage [33,34,35,36,37,38,39].

Considering the similarities between MDD and PCD symptoms [40], another argument in favor of the significant role of demyelination in PCD is that MDD is often considered a disorder of WM connectivity, which is caused by changes in brain myelination. Impaired WM integrity and demyelination in patients with MDD have been shown in several neuroimaging studies [41,42,43,44,45,46,47].

Despite the large number of publications related to brain demyelination in post-COVID-19 patients, we have not encountered a single study that systematically assessed demyelination in patients with diagnosed clinical depression [36]. In this work, we applied the quantitative MRI method called macromolecular proton fraction (MPF) mapping to assess brain demyelination as a possible cause of post-COVID depression. MPF mapping is standing out among other MRI methods sensitive to myelin due to its improved specificity to myelin and fewer physiological confounders based on diffusion, relaxation, and susceptibility [48,49,50]. MPF has the strongest correlations with myelin content assessed with histology [51,52,53,54,55]. MPF values are independent of the magnetic field strength and provide a solid method for myelin measurements across a variety of human and animal MRI platforms [56,57]. Finally, MPF maps can be obtained without modification of the original manufacturers’ pulse sequences available on the standard clinical MRI scanners [58,59,60], including for assessing demyelination in mental disorders [58].

The aim of the current study was to quantify the degree of brain myelination in patients with clinically diagnosed post-COVID depression. We hypothesize that depression in the post COVID-19 period is directly related to the decrease in brain myelination.

## 2. Materials and Methods

### 2.1. Study Participants

Eighty seven participants were recruited by Tomsk State University (Tomsk, Russia), the Mental Health Research Institute (Tomsk, Russia), and the Medical Diagnostic and Treatment Center (Tomsk, Russia) between September 2022 and June 2023. The inclusion criteria were the following: age from 18 to 60 years, previous positive COVID-19 PCR test, and persistence of post-COVID complications (except for the control group). The exclusion criteria were: the history of traumatic brain injury, diagnosed neurological or psychiatric condition prior to COVID-19 infection, pregnancy, symptoms of acute infectious and somatic diseases, contraindications to MRI, inability to tolerate the MRI procedure, and self-withdrawal from the study. Written, informed consent was obtained from all participants. The study design was approved by the local Ethical Committee of the Mental Health Research Institute (protocol №15 dated 25 August 2022) and the Bioethics Committee of Tomsk State University (№12 dated 6 June 2022), following the guidelines of the Declaration of Helsinki.

Patients with post-COVID complications were recruited by a neurologist at the Medica Diagnostic and Treatment Center and a psychiatrist at the Mental Health Research Institute. The control group was formed from employees and students of Tomsk State University, as well as their relatives and friends, who had no prior COVID-19 history. Patients and healthy volunteers were interviewed regarding eligibility for inclusion and exclusion criteria. Those who met these criteria signed an informed consent form and completed the questionnaire (Supplementary Materials). A few days later, participants underwent an MRI scan. Data acquired from five participants who were newly diagnosed with brain pathologies based on MRI (cavernoma, glioma, vascular anomalies) were excluded from further analysis.

All patients with complications after COVID-19 were examined by a psychiatrist to establish or exclude a diagnosis of clinical depression. The PCD group was formed from the patients with post-COVID complications who met the clinical criteria for depression (moderate depressive episode—F32.1, severe depressive episode without psychotic symptoms—F32.2, recurrent depressive disorder (first diagnosed at the time of the study): current episode moderate—F33.1, according to ICD-10), and the symptoms of depression appeared after the coronavirus infection and were absent before it. A group of patients with PCD was formed by a psychiatrist based on a semi-structured psychiatric interview corresponding to the list of symptoms of mental disorders in the International Statistical Classification of Diseases and Related Health Problems (ICD-10) [61] and medical history, socio-demographic characteristics, the COVID-19 questionnaire’s data, and psychometric assessment. The severity of depressive symptoms was assessed using two scales: the Hospital Anxiety and Depression Scale (HADS) and the Hamilton Depression Rating Scale (HDRS) [62]. The screening testing with HADS was carried out by a clinical psychologist. The HDRS testing was carried out by a qualified clinical psychiatrist during a clinical interview. After that, the participants who had a HADS score > 8 were assessed by a psychiatrist. The severity of the current depressive episode was assessed before the start of drug therapy using the Hamilton Rating Scale for Depression (HDRS) [63,64]. The total score is interpreted as follows: no depression (0–7); mild depression (8–16); moderate depression (17–23); and severe depression (≥24).

The post-COVID depression (PCD) group included 25 individuals with first diagnosed clinical depression. The comparison group (noPCD group, n = 38) included participants with neurological complications of COVID-19 and without clinical depression. The control group (n = 19) consisted of healthy volunteers who were not COVID-19 positive and did not experience symptoms of COVID-19 during the entire pandemic until the time of examination. The demographic characteristics of participants are presented in Table 1. The groups did not differ significantly in age, gender, vaccination status at the time of the study, or severity of COVID-19 (PCD and noPCD groups) according to Chi-square criteria. The percentage of patients in the PCD and noPCD groups vaccinated before the first episode of COVID-19 was significantly lower than in the control group. There were n differences in the percentage of PCD and noPCD patients vaccinated before the first COVID-19 episode.

### 2.2. Patient Survey

The COVID-19 questionnaire was filled out by the study participants (Supplementary Materials). The questionnaire included questions about the symptoms of the acute and post-COVID phases, the PCR tests, vaccination status, the number of COVID-19 episodes, their dates, and their severity. As symptoms of the acute phase, patients noted the presence or absence of anosmia, ageusia, fever, difficulty breathing, cough, muscle weakness, myalgia, headache, and dizziness. As symptoms of the post-COVID phase, patients noted the presence or absence of headache, dizziness, brain fog, anosmia, ageusia, sensitivity, hypertensia/hypotensia, insomnia, fatigue, attention and memory deficit, myalgia, depression, and panic attacks. The number of symptoms in the acute and post-COVID phases was calculated as the sum of symptoms (1 symptom–1 score; maximum scores were 9 for acute symptoms and 14 for post-COVID symptoms), to which positive answers were given for all diseases. The number of symptoms was previously shown as a predictor of post-COVID complications and for assessing the severity of post-COVID [65,66,67,68].

### 2.3. MRI Data Acquisition

All participants underwent magnetic resonance imaging using the 1.5T clinical scanner Magnetom Essenza (Siemens, Erlangen, Germany). The imaging protocol for obtaining MPF maps [58] included three 3D-spoiled gradient-echo pulse sequences. The following acquisition parameters were used:

- Magnetization-transfer (MT)-weighted pulse sequence: TR = 20 ms, echo time (TE) = 4.76 ms, flip angle (FA) = 8°, scan time 5 min 40 s;
- T1-weighted pulse sequence: TR = 16 ms, TE = 4.76 ms, FA = 18°, scan time: 4 min 32 s;
- Proton-density (PD)-weighted pulse sequence: TR = 16 ms, TE = 4.76 ms, FA = 3°, scan time: 4 min 32 s.
- The additional imaging sequences included the following:
- 3D Fluid attenuated inversion recovery (FLAIR) pulse sequence: TR = 5000 ms, TE = 390 ms, TI = 1800 ms;
- 3D T1-weighted pulse sequence: TR = 16 ms, TE = 4.76 ms;
- 3D T2-weighted pulse sequence: TR = 3000 ms, TE = 335 ms.

All scans were acquired in the sagittal imaging plane with a voxel size of 1.25 × 1.25 × 1.25 mm^3^, a field of view of 240 × 240 × 200 mm^3^, a matrix of 192 × 192 × 160, and single signal averaging. 

The total time of scanning per subject was about 35 min.

### 2.4. Image Processing

MPF maps were reconstructed using a single-point algorithm with a synthetic reference image [69,70] implemented in the previously developed software available at https://www.macromolecularmri.org/ (assessed on 1 June 2024).

**Figure 1:**
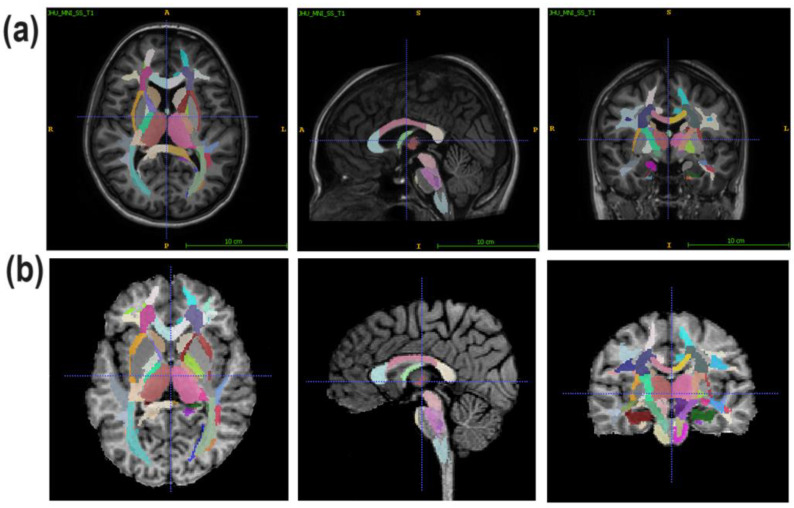
An example of the segmentation of an individual MPF map (**b**) using its registration to the T1 Eve template (**a**) Reprinted with permission from [73]. 2009, Oishi et al. Slices in similar axial, sagittal, and coronal projections are shown.

Regional WM and GM segmentation was performed using Advanced Normalization Tools (ANTs) [71,72], version v.2.4.4 and Eve anatomical atlas [73], as described in [74]. The T1 template image of Eve atlas and individual MPF maps were co-registered using the antsRegistrationSyNQuick algorithm. To register the template atlas labels to individual MPF maps (Figure 1), the obtained deformation field was applied to Type-III Eve atlas segmentation [73].

The measurements of 118 GM and WM brain structures located in the right and left hemispheres were performed using MPF maps and ITK-snap, version 3.6.0, software. The list of investigated structures is as follows:

1. Juxtacortical (superficial) WM: superior parietal, superior, middle, and inferior frontal; lateral and middle fronto-orbital; rectus; precentral; postcentral; angular; pre-cuneus; cuneus; lingual; fusiform; superior, inferior, and middle occipital; superior, inferior, and middle temporal; supramarginal; the cingulum (parts of the cingulate gyrus and hippocampus);
2. WM pathways and fasciculi: corticospinal tract (CST); anterior, superior, and posterior corona radiata (CR); anterior limb, posterior limb, and retrolenticular part of internal capsule (IC); genu, body, and splenium of corpus callosum (CC); medial lemniscus; inferior, superior, and middle cerebellar peduncles (CPs); cerebral peduncles; posterior thalamic radiation; fornix (FX) (stria terminalis, column, and body); superior longitudinal (SL) fasciculus; superior (SFO) and inferior fronto-occipital (IFO) fasciculi; uncinate fasciculus; sagittal stratum; external capsule; pontine crossing tract; tapetum;
3. Subcortical and allocortical GM structures: amygdala; caudate nucleus; putamen; globus pallidus; hippocampus; entorhinal area; thalamus;
4. Brainstem structures: medulla; pons; midbrain.

For the midbrain, pons, and medulla, the measurements in the left and right hemispheres were averaged. Other brain structures were analyzed, taking into account the hemisphere to which they belonged.

### 2.5. Statistical Analysis

Statistical analysis was carried out using Statistica 10.0 software. Differences in MPF between the PCD, noPCD, and control groups for each brain structure were analyzed using the repeated measures analysis of variance (ANOVA), followed by post hoc Fisher LSD tests. In the analyses of multiple brain structures, *p*-values were adjusted using the Benjamini–Hochberg procedure for false discovery rate (FDR) correction to prevent false positive results in multiple comparisons. The FDR level was set to 0.05. A one-way ANOVA was used to assess between-group differences in MPF of brainstem structures, results of psychological tests, and parameters associated with COVID-19, such as severity, time after recovery, and the number of acute and post-COVID symptoms. 

Multiple regression and logistic regression analyses were performed to identify the best predictors of clinical post-COVID depression. First, for all patients in the PCD and noPCD groups for all studied brain structures, the percentage change in MPF relative to the control group was calculated using the formula: (MPF_ind_ − MPF_control_)/MPF_control_ × 100, where MPF_ind_ is the individual MPF value for each brain structure MPF_control_ is the average MPF for the same structure in the control group. A total of 115 variables were obtained, corresponding to the percentage changes in the MPF of 59 structures in the left and right hemispheres. To avoid errors associated with multicollinearity of data for multiple regression analysis, the values of percentage changes in MPF were examined using factor analysis. Velicer’s Minimum Average Partial (MAP) test, recommended for relatively small samples [75], was used to determine the number of factors for subsequent analyses. As a result, principal component analysis allowed us to identify nine independent factors that explained 75.3% of the variance in total. To interpret the obtained factors, the percentage changes in the MPF of structures with factor loadings > 0.5 were considered. The individual factor scores were used for multiple regression analyses. The multiple regression analysis was performed for the PCD and noPCD groups separately and for the total sample of post-COVID patients. The HDRS score was used as the dependent variable. Independent variables included nine variables: individual factor scores, age, gender, time since the acute phase of the first and last disease, and the number of symptoms of the acute and post-COVID phases. The quality of regression models was assessed using the multiple R correlation coefficient and the R^2^ determination coefficient. The contribution and significance of predictors were assessed using beta-coefficients and *p*-values.

Logistic regression analysis was performed to clarify the variables that allow for the prediction of the presence or absence of clinical depression in patients with post-COVID complications. The same variables as in the multiple regression analysis were included as independent variables in the logistic regression analysis. A categorical variable corresponding to the division of patients into PCD and noPCD groups was included as a dependent variable. The goodness of fit of regression models was assessed using the Akaike Information Criterion (AIC), Bayesian Information Criterion (BIC), logit likelihood, odds ratio, and percentage of correctly predicted cases. The contribution and significance of predictors were assessed using the significance of the Wald statistic and logit likelihood.

All tests were considered statistically significant with *p* values less than 0.05.

## 3. Results

### 3.1. Acute and Post-COVID Symptoms

Group characteristics related to disease severity, time since the first and last COVID-19, and symptoms in the acute and post-COVID phases are shown in Table 2.

Patients in the PCD group with depression differed significantly from the noPCD group on several symptoms in both the acute and post-COVID phases. In the acute phase, the PCD patients more often than the noPCD noted ageusia, cough, and headache; however, these differences were on the borderline of statistical significance. The total number of acute symptoms in the PCD group was significantly higher than in the noPCD group (*p* = 0.008). The differences between the groups were even more essential in the post-COVID phase. More than half of the PCD patients experienced anosmia and ageusia (64% and 56%, respectively), while in the noPCD group, only 29 and 21% of patients reported these symptoms. About half of the patients in the noPCD group reported sleep disturbances, fatigue, attention deficits, and depression. In contrast, almost all patients in the PCD group reported these symptoms. The average number of post-COVID symptoms in the PCD patients is 1.7 times higher than in the patients in the noPCD group (*p* < 0.0001) (Table 2).

### 3.2. Neuropsychological Results

Table 3 demonstrates the results of the neuropsychological testing of the study participants based on their medical diagnosis.

As expected, patients with clinical post-COVID depression showed significantly higher scores on the HDRS and HADS depression-related scales, not only compared to controls but also compared to the noPSD group. Hamilton scores in the PSD group were 4.6 times higher than in the control group and 3.0 times higher than in the noPSD group. The total HADS score for the PSD group was 2.7 times higher than in the control group and 2.3 times higher than in the noPSD group. The noPSD group also showed significantly higher HDRS and HADS scores in comparison with controls. The noPSD group showed significantly higher HDRS and HADS scores compared to the control group, but the differences between groups were smaller (1.5 and 1.2 times, respectively).

### 3.3. Brain Demyelination in Patients with Post-COVID Depression

**Figure 2:**
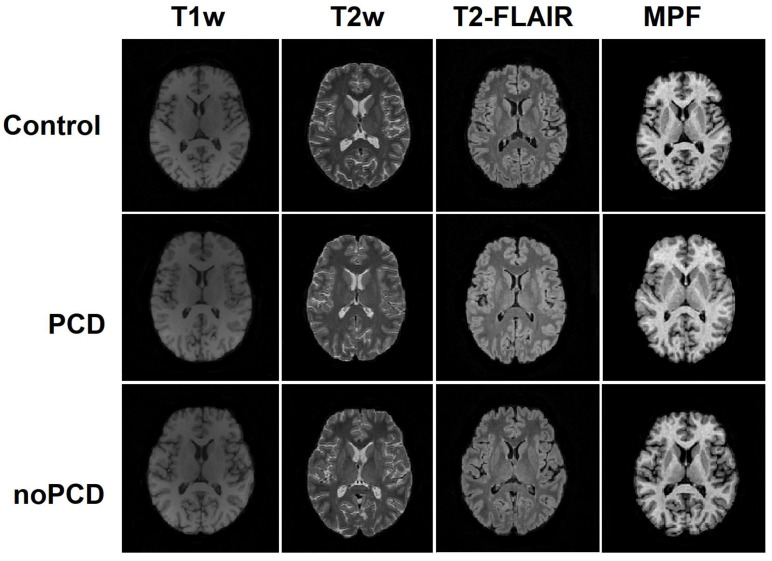
Example MPF maps, T1, T2, and T2-FLAIR images of the participants from the PCD (44 years, female), noPCD (48 years, male), and control (44 years, female) groups.

Brain images obtained from the study participants using multiple MRI techniques did not show visible differences between the healthy control, PCD, and no-PCD groups. Example MPF maps, T1w, T2w, and T2-FLAIR images of the participants of similar age from all groups are shown in Figure 2.

**Figure 3:**
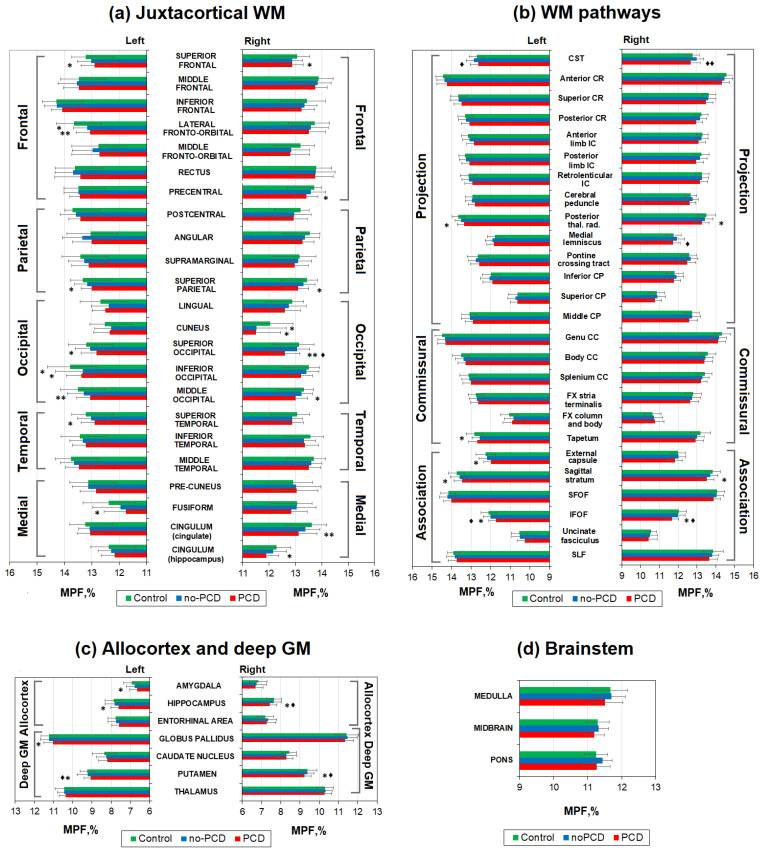
Differences in MPF measurements between the PCD, noPCD, and control groups for the separate brain regions: (**a**) juxtacortical WM, (**b**) WM pathways, (**c**) allocortex and deep GM, (**d**) brainstem. Significant differences relative to controls: * *p* < 0.05, ** *p* < 0.01. Significant differences relative to the noPCD group: ♦ *p* < 0.05, ♦♦ *p* < 0.01. Error bars correspond to SD.

**Figure 4:**
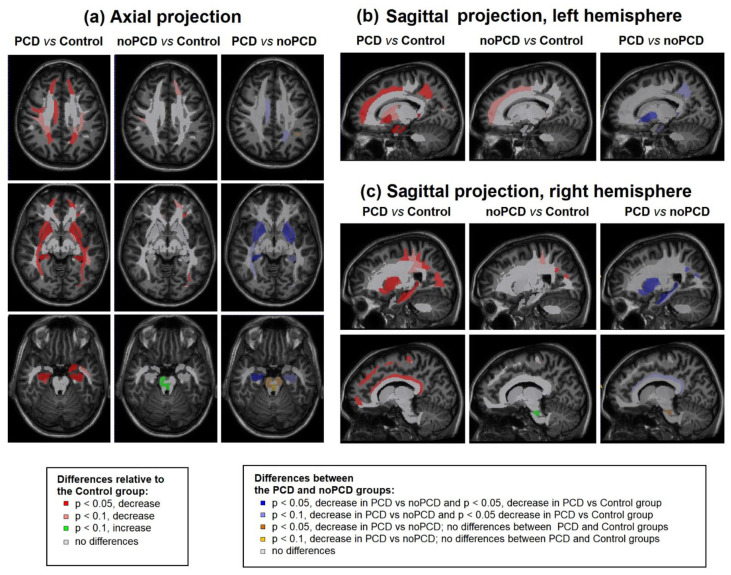
Examples of the brain slices of Eve template (Reprinted with permission from [73] 2009, Oishi et al.) that illustrate significant (*p* < 0.05) and near significant (*p* < 0.1) differences between groups at the levels of superior juxtacortical WM, basal ganglia, and brainstem of axial views (**a**) and sagittal views of the left (**b**) and right (**c**) hemispheres.

Quantitative MPF mapping was more sensitive in detecting differences in myelination of brain structures in study participants. Figure 3 demonstrates a significant decrease in myelination in a large number of WM as well as GM brain structures and brainstems in the PSD group in comparison to the control and the noPSD groups. Figure 4 shows percentage differences in MPF for the PCD and noPCD groups relative to the level of controls. Specifically, a significant myelination decrease was observed in the juxtacortical regions of the WM (Figure 3a). The regions of the occipital and frontal lobes, as well as the regions of the medial brain surface, were most affected. A 2–4% decrease in MPF was observed in all parts of the occipital lobe (inferior, superior, and middle occipital WM) of both hemispheres in the PSD group compared to controls. Inferior occipital WM also showed a decrease in the myelination the PSD group relative to the control group. For the frontal lobe, a significant decrease in MPF in the PCD group compared to controls was found for the superior frontal WM of both hemispheres, the lateral fronto-orbital WM of the left hemisphere, and the precentral WM of the right hemisphere. A significant myelination decrease in the PCD group compared to controls was found for the superior parietal WM of both hemispheres and the left superior temporal WM. In addition, a significant demyelination in the PCD group compared to controls was found for the medial surface: right cuneus, left cingulate, and fusiform WM. For the lateral fronto-orbital and cuneus WM, a decrease in MPF was found in the noPCD group, although of a smaller magnitude than in the PCD group (3.3% and 4.2%, respectively, Figure 4a). Among all juxtacortical structures, the largest decrease in MPF (5%) was found for the fusiform WM (medial part of juxtacortical WM).

Subcortical WM pathways, including association, projection, and commissural, also revealed a significant decrease in myelination in the PCD group compared to the control group (Figure 3b). The most prominent demyelination was observed for the association WM pathways. The inferior fronto-occipital fasciculus (IFOF), sagittal stratum of both hemispheres, and left external capsule showed a significant decrease for the PCD group compared to controls. Additionally, the CST of both hemispheres and the right medial lemniscus showed a significant PMF decrease for the PCD group compared to the noPCD group. A decrease in MPF was also observed in the projection WM pathway and the posterior thalamic radiation of both hemispheres in the PCD patients compared to controls. Among the commissural WM pathways, only the left tapetum showed a significant decrease compared to controls. Among all WM pathways, the greatest MPF reduction was observed for IFOF (3%, Figure 4b), the association WM pathway. No significant differences in myelination were observed in WM pathways between the noPCD comparison group and controls.

A decrease in myelination was also observed in the allocortex and deep GM structures of the PSD cohort (Figure 3c) in comparison to the controls and noPSD group. The MPF decrease was observed in the hippocampus and putamen of both hemispheres, as well as in the amygdala and globus pallidus of the left hemisphere. The largest MPF differences of 3% and more were found in the amygdala and hippocampus, which are part of the allocortex (Figure 4c).

A few brain structures of the PCD cohort patients exhibited differences in MPF with both noPCD and control. Specific structures are the putamen, the IFOF of both hemispheres, the right hippocampus, and the superior occipital WM. For the CST of both hemispheres and the left medial lemniscus, a decrease in MPF was observed for the PCD group compared to the noPCD group, with no significant differences from the control.

Brainstem structures did not reveal any significant differences between groups (Figure 3d).

**Figure 5:**
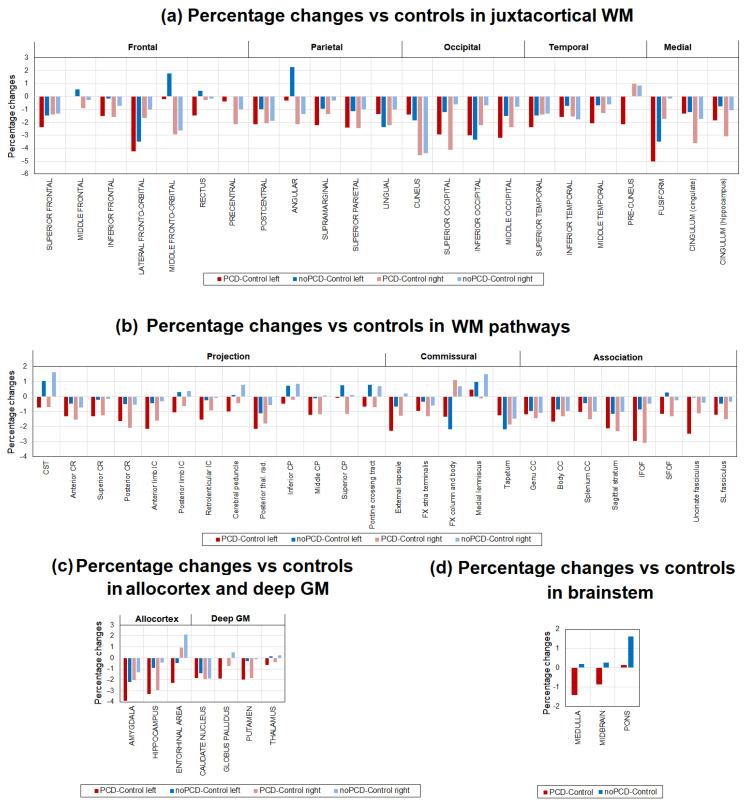
The percentage changes between MPF measurements obtained in the PCD, noPCD, and control groups are (**a**) juxtacortical WM, (**b**) WM pathways, (**c**) allocortex and deep GM, and (**d**) brainstem.

Figure 5 was built based on the Eve anatomical atlas, which was used for segmentation, and indicates the regions for which significant differences in MPF were obtained. It shows the spatial differences for each ROI between groups, shown in detail in Figure 3. Significant (*p* < 0.05) or near significant (*p* < 0.1) differences in myelination are shown between groups at the levels of superior juxtacortical WM, basal ganglia, and brainstem of axial views (Figure 5a) and sagittal views of the left (Figure 5b) and right (Figure 5c) hemispheres. Extensive demyelination was detected in PCD patients compared to controls. The reduction in myelination was less extensive in the noPCD group compared to controls. A significant decrease in MPF was observed in a large number of GM and WM brain structures in the PCD group compared to noPCD and controls. A significant or nearly significant decrease in myelination was observed only in the brainstem structures of the PCD group compared to noPCD, in the absence of differences with controls.

### 3.4. Specificity of Demyelination in Patients with Post-COVID Depression

Multiple regression analysis was used to identify brain structures where changes in myelination are closely related to the manifestation of clinical post-COVID depression. A factor analysis was applied to exclude multicollinearity of independent variables–potential predictors of multiple regression. As a result, nine factors were identified that explained 75% of the MPF variability in the post-COVID patients (Table 4). The factors that could be uniquely linked to the specific brain structures were examined further. The identified factors did not correlate with each other, which confirmed their unique contribution to the data variance.

The nine factors associated with the percentage change of MPF, along with age, gender, disease severity, time since recovery, and the number of symptoms in the acute and long phases, were included in multiple regression analyzes as potential predictors of Hamilton scores. The results of the analysis are shown in Table 5. Factor 7, associated with IFOF and uncinate fasciculus, was identified as the significant predictor of HDRS scores for the total sample of patients. (Table 4). The number of post-COVID symptoms was significant in the regression equation for the total sample and noPCD groups. The number of acute symptoms was significant in the regression equation only for the PCD group. 

Table 6 shows the results of the logistic regression analysis. Model 1 includes the number of post-COVID symptoms and factor 7 as significant predictors of the presence of clinical depression in patients with post-COVID complications. According to the analysis, model 1 allowed for the correct classification of 60% of patients in the PCD group and 78% of patients in the noPCD group. The goodness of fit of this model (less AIC and BIC) was the best among all models built via logistic regression, excluding Model 2, which included an additional gender factor (Table 6). According to this model, female sex is a significant predictor of depression in post-COVID patients. Model 2 has a higher odds ratio, a lower AIC, and a higher percentage of correctly predicted classifications (68% for the PCD group and 87% for the noPCD group).

The addition of interactions between MPF-related and other variables did not improve the parameters of the models built using multiple and logistic regression analyses.

## 4. Discussion

In this work, brain demyelination was investigated as a possible cause of post-COVID depression. Patients with newly diagnosed post-COVID-19 clinical depression showed extensive brain demyelination. Changes in myelination were statistically significant in comparison with the post-COVID patients with depression (PCD) and controls without previous COVID-19, as well as in comparison with the patients with long-term complications after COVID-19 but without diagnosed depression. Patients in the PCD group showed extensive demyelination of the juxtacortical WM, most pronounced in the occipital lobe but also including the frontal, parietal, and temporal lobes. In addition, patients in this group showed demyelination of the WM tracts, the most prominent of the association pathways, including the IFOF, sagittal stratum, and left external capsule. Projection (posterior thalamic radiation) and commissural (left tapetum) WM pathways were also affected. Additionally, we found GM demyelination, including the hippocampus, putamen, left globus pallidus, and amygdala. Significant demyelination was also observed in the noPCD group compared to the control group, but with a lower magnitude and a smaller affected area than in the PCD group.

Multiple regression and logistic regression analyses revealed only one factor as the main predictor of post-COVID depression, factor 7, which was related to IFOF and the uncinate fasciculus measurement in both hemispheres. This factor was included as a significant predictor in both the linear regression equation for the total sample, which differentiates the PCD and noPCD groups (R^2^ = 0.41, *p* < 0.001), and in the regression equation predicting the severity of depression by Hamilton score in the PCD group (R^2^ = 0.68, *p* < 0.001). Moreover, factor 7, along with the number of post-COVID symptoms, was found to be a significant predictor of clinical depression in the logistic model. The patients in the PCD group showed significant demyelination in the IFOF compared with both the non-PCD group and the control, according to ANOVA results. The uncinate fasciculus showed only a trend (*p* = 0.08) toward decreased MPF for the PCD group compared with the noPCD group and no significant differences with the control group. Thus, according to the results of three types of analysis, IFOF demyelination can be considered the best predictor of clinical post-COVID depression.

The number of symptoms in the acute and post-COVID phases was also important in group classification. The equations for the total sample and noPCD group included the number of symptoms in the post-COVID phase, and this variable was the only significant predictor for the noPCD group. In contrast, the regression equation for the PCD group included the number of symptoms in the acute phase.

Self-reported depression might have affected the results of multiple linear and logistic regressions. However, the question about the presence of depression was one out of 14 self-reported post-COVID symptoms, accounting for only 7% of the number of symptoms in the survey. At the same time, the average number of symptoms in the PCD group was 40% less than in the noPCD group (8.04 and 4.88, respectively). Therefore, the percentage of self-assessed depression among all post-COVID symptoms can be considered negligible. Additionally, the self-assessment of depression in the questionnaire is most likely to be related to mood changes reported by the study participants. There is a fundamental difference between the self-assessment of depression and the clinical diagnosis of this condition made by a professional psychiatrist. While 34% of patients in the noPCD group self-reported depression in the questionnaire, the psychiatrist found no signs of MDD in those patients.

Gender was included in one of the two best logistic models predicting the presence of PCD. Specifically, the female gender was identified as a predictor of the presence of PCD. This result is not surprising given the fact that female gender is the risk factor for long-term neurological consequences of COVID-19 [65,68,76,77,78,79], as well as for developing MDD [80,81]. The increased risk of post-COVID complications in women may be based on gender-specific differences in the autoimmune response [82,83]. Our recent study revealed sex differences in the cognitive performance of post-COVID patients with and without depression [84].

According to the literature, depression is often considered a disorder of WM connectivity [41,42,43,44,45,46,47] in which IFOF plays a significant role [45,46,47]. The IFOF connects early visual processing in the cuneus and lingual gyrus as well as parts of the parietal lobe to frontal lobe regions and plays a critical role in semantic language processing, goal-oriented behavior, and visual switching tasks [85,86]. In addition, the tract includes the connections between the cingulo-opercular and frontoparietal networks related to executive function and goal-oriented behavior [86,87]. High-angle diffusion spectrum imaging analysis identified five subcomponents of the IFOF, which primarily included connections from the frontal or fronto-orbital cortex to the inferior, superior, and middle occipital lobes [88]. The IFOF degeneration has been demonstrated in patients with Alzheimer’s disease and neuropsychological behavioral disorders, including antisocial personality disorder and obsessive compulsive disorder [89,90,91].

Demyelination of IFOF along with other brain structures has been described in MDD patients [45,47,92,93]. Lai et al. [45] found lower fractional anisotropy (FA) in the bilateral IFOF, SLF, inferior longitudinal fasciculi, and CC in MDD patients compared to controls. Changes in FA were found in the left IFOF, uncinate fasciculus, anterior thalamic radiation, and bilateral CC compared to the patients with panic disorder. Liang et al. [93] identified three subgroups of MDD patients based on the spatial localization of reduced FA: the first group with widespread WM disruption (decrease in 8 of 20 studied tracts, including IFOF), the second group with a predominant decreased FA in the CC and left cingulate, and the third group with no statistically significant tract disruption. Reduced FA in the genus of the corpus callosum, IFOF, and posterior thalamic radiation in MDD patients was found by Coloigner et al. [47].

Different studies reported different numbers of WM-demyelinated brain structures in MDD patients [42,43,44,45,47,92,93,94,95]. Thus, Reppermund et al. [96] found a significant decrease in FA in 45 brain regions, while the study by Hollocks et al. [97] found no significant association between our WM parameters and depressive symptoms. Demyelination in MDD patients was observed most often in the CR [42,98], IFOF [42,45,47,92,93,98], uncinate fasciculus [45,98], posterior thalamic radiation [47,96,99], cingulum [42,93,95,98], sagittal stratum [42,43,98], IC [42,43,47], and frontal lobe [44,96], which was confirmed by our results. Other demyelinated brain structures in MDD were CC [42,45,47,93,94,96], SLF [96,98,99], and FX [42,43,94], in which we did not find significant changes compared to controls. Perhaps the reason for these discrepancies lies in the differences in the etiology of MDD and post-COVID depression.

The etiology of MDD is commonly considered multifactorial; in other words, it might be caused by the interaction of biological, genetic, environmental, and psychosocial factors [19]. The results of our current study point to COVID-19 as the main factor causing the recent depressive episode. The multiple regression results identified the number of symptoms in the acute and post-COVID phases as significant predictors of the presence and severity of clinical depression. Patients in the PCD group were significantly more likely to report ageusia, cough, and headache in the acute phase, as well as anosmia, ageusia, insomnia, fatigue, and attention deficit in the post-COVID phase, compared to patients in the noPCD group. At the same time, factors such as age, gender, and severity of COVID-19 were not among the significant predictors of post-COVID depression, although previous studies have shown these factors as predictors of post-COVID complications [65,68,76,77,78,79].

In addition to WM demyelination, we found a decrease in MPF in PCD patients for GM structures: hippocampus, left amygdala, putamen, and left globus pallidus. Since all published studies measured only WM myelination, we cannot compare our results describing demyelination of GM brain structures in MDD patients, although some evidence suggests the involvement of the amygdala, hippocampus, and deep GM in depressive disorders [42,100]. Application of MPF mapping to myelin quantification was an important advantage of our study in comparison with data published with the use of DTI and other MRI methods. MPF mapping allows reliable quantification of weak GM myelination [51,52,54,55,58] and is independent of iron accumulation in the basal ganglia [101].

Several possible pathological mechanisms for demyelination development as a consequence of COVID-19 were described in detail in our recent review [6]. Key factors include inflammation, the direct effects of the virus on oligodendrocytes, and cerebrovascular disorders that cause myelin damage. The acute phase of COVID-19 is accompanied by a cytokine storm phenomenon, accompanied by high levels of pro-inflammatory cytokines in the blood serum [102] that cause an overactive immune response [22,103]. Cytotoxic T lymphocytes and proinflammatory cytokines can cross the blood–brain barrier, activating macrophages, microglia, and astrocytes and causing immune-mediated demyelination [104,105,106]. In addition, high titers of autoantibodies have been shown in post-COVID patients, which correlates with the severity of the disease [107]. Some of these antibodies attack components of the myelin sheath [108,109,110]. High levels of cytokines and an autoimmune response are observed not only in the acute phase but can persist for a long time after COVID-19 [109,110]. Since SARS-CoV-2 can cross the blood–brain barrier [111], another cause of demyelination may be related to direct infection of oligodendrocytes, leading to dysfunction and cell death [112,113]. Finally, COVID-19-associated decreased lung function and respiratory depression lead to hypoxia, thromboembolism with microbleeds, and cerebral ischemia [4,114,115,116]. In turn, impaired cerebral blood flow causes the death of oligodendrocytes, which are extremely sensitive to oxygen and glucose deficiency [117,118]. Myelin destruction follows the death of neurons and axons [54], caused by ischemia.

A few studies systematically examined myelination in post-COVID patients [33,34,35,36,37,38,39]. The works by Huang et al. examined WM changes in the longitudinal MRI (DTI, DKI, and NODDI) studies one [34] and two [33] years after COVID-19 recovery. They found abnormal diffusion metrics in the corona radiata, genus of the CC, and left SLF in one year after recovery, and in the CC, CR, CP, IC, posterior thalamic radiation, sagittal stratum, left external capsule, SLF, and CST in two years after recovery. Lower FA in the body of the corpus callosum was observed in the acute phase of COVID-19. Inflammation levels in the acute stage are positively correlated with white matter abnormalities and negatively with cognitive function. Qin et al. [37] revealed significant changes in the volumes of numerous WM structures and in FA in severe compared to mild patients and in mild patients compared to control patients. FA differences were found in the following WM tracts: anterior thalamic radiation, SLF, optic radiation, ILF, inferior longitudinal fasciculus, forceps minor, right IFOF, left FX, acoustic radiation, cingulum, and frontal aslant tract. An MRI study by Bispo et al. [35] in patients about 3 months after COVID-19 recovery showed no changes in GM and lower fiber-specific apparent fiber density in the corona radiata, CST, CC, arcuate fasciculus, cingulate, fornix, IFOF, inferior longitudinal fasciculus, SLF, and uncinate fasciculus. Thus, there was a significant overlap between our results and the literature data regarding a number of brain structures affected by the disease, in particular the IFOF, cingulum, corona radiata, IC, posterior thalamic radiation, sagittal stratum, external capsule, and uncinate fasciculus. The differences in results can be explained by the variability in COVID-19 complications among patients.

We found only one work by Benedetti et al. [36] that examined associations between the manifestations of post-COVID depression, brain myelination, and functional connectivity. The study included voxel-based morphometry, DTI, and resting-state fMRI on 42 patients imaged 3 months after COVID-19. Self-rated depression was inversely correlated with GM volumes in the anterior cingulate cortex and insula, axial diffusivity, and functional connectivity. In this study, depressive psychopathology was self-rated on the Zung Self-Rating Depression Scale, and high scores (>9) were observed in only 9 of 42 patients. This situation is critically different from our study of a homogeneous group with a clinical diagnosis of depression made by a psychiatrist. Unfortunately, in the study by Benedetti et al., the control group was missing. Our results partially overlap with the above in terms of decreased connectivity in singular WM, but differences in samples and the lack of a control group do not allow us to draw a clear conclusion.

## 5. Conclusions

To date, very few studies have been carried out on changes in myelination in post-COVID complications and, in particular, in post-COVID depression. The present study is the first to show widespread demyelination in WM and GM brain structures in patients with clinically diagnosed depression caused by COVID-19. These changes were observed both in comparison with controls and in comparison with patients with post-COVID complications but without diagnosed depression. The IFOF has been identified as a key structure where the presence of demyelination is the best predictor of the presence and severity of post-COVID depression. Future studies will clarify whether IFOF demyelination is a specific feature associated with the effects of SARS-CoV-2 infection or is a general feature of depressive disorders.

## 6. Study Limitations

Our study is limited by using the age range of 18 to 60; older individuals with PCD were not studied. Another important limitation is the relatively small sample size. Undoubtedly, MRI studies with a very large sample size, like the work of Marek et al. [119], with a sample of about 50,000 individuals, lead to more reliable conclusions. We did not perform serological tests for antibodies against SARS-CoV-2 in the blood plasma of study participants. The vaccination status of the PCD and noPCD before the first episode of COVID-19 differed from that of the control participants. Although all patients in the PCD group were first diagnosed with clinical depression after COVID-19, we cannot guarantee that its occurrence was not influenced by socio-demographic factors such as isolation, negative information flow, losses, economic instability, and decreased quality of life. There are only two psychiatric scales, HDRS and HADS, for assessing the severity and symptoms of depression. Testing with other psychiatric scales, as well as surveys of family members and caregivers, may provide additional objective information about symptoms of depression [120]. Future studies in other age ranges, using other MRI modalities in combination with testing for depressive symptoms, will help to better understand the features and mechanisms of post-COVID depression.

## Figures and Tables

**Table 1:** The demographic characteristics of participants of the study.

Parameter	PCD	No-PCD	Control
Sample size	25	38	19
Male (%)	4 (16)	14 (29)	8 (42.1)
Female (%)	21 (84)	24 (71)	11 (57.9)
Age, years ± SD	36.96 ± 13.7	42.05 ± 9.17	38.53 ± 10.57
Age, median (min-max)	42 (19–59)	42 (21–58)	37 (20–56)
Vaccinated before the first COVID-19 episode (%)	10 (40) *	9 (23.7) ***	14 (73.7)
Vaccinated at the time of the study (%)	20 (80)	24 (63)	14 (73.7)

Significant differences in comparison to the control group: * *p* < 0.05, *** *p* < 0.001.

**Table 2:** The severity of COVID-19, the number of COVID-19 episodes, and the time after recovery. acute and post-COVID symptoms of participants in the study, according to the health questionnaire.

Parameter	PCD	noPCD	Statistics
Severity, mild/moderate/severe (%)	88/8/4	73/24/3	F(2, 79) = 1.74, *p* = 0.18
Number of COVID-19 episodes, mean ± SD	1.6 ± 0.7	1.5 ± 0.7	F(1, 61) = 0.33, *p* = 0.56
Time after the first COVID-19, months ± SD	20.3 ± 8.2	19.7 ± 9.8	F(1, 61) = 0.06, *p* = 0.81
Time after last COVID-19, months ± SD	13.1 ± 10.3	13.8 ± 9.9	F(1, 61) = 0.07, *p* = 0.79
**Acute symptoms**			
Anosmia, n (%)	22 (88%)	29 (76%)	Chi sq, *p* = 0.25
Ageusia, n (%)	19 (76%) *	19 (50%)	Chi sq, *p* = 0.04
Fever, n (%)	22 (88%)	36 (95%)	Chi sq, *p* = 0.33
Difficulty breathing, n (%)	14 (56%)	17 (45%)	Chi sq, *p* = 0.38
Cough, n (%)	22 (88%) *	24 (65%)	Chi sq, *p* = 0.04
Muscle weakness, n (%)	24 (96%)	35 (92%)	Chi sq, *p* = 0.53
Myalgia, n (%)	20 (80%)	22 (58%)	Chi sq, *p* = 0.07
Headache, n (%)	22 (88%) *	25 (66%)	Chi sq, *p* = 0.047
Dizziness, n (%)	14 (56%)	15 (39%)	Chi sq, *p* = 0.20
Number of acute symptoms	7.24 ± 1.85 **	5.82 ± 2.13	F(1, 61) = 7.45, *p* = 0.008
**Post-COVID symptoms**			
Headache, n (%)	7 (28%)	4 (11%)	Chi sq, *p* = 0.07
Dizziness, n (%)	10 (40%)	13 (34%)	Chi sq, *p* = 0.64
Brain fog, n (%)	14 (56%)	16 (42%)	Chi sq, *p* = 0.28
Anosmia, n (%)	16 (64%) **	11 (29%)	Chi sq, *p* = 0.006
Ageusia, n (%)	14 (56%) **	8 (21%)	Chi sq, *p* = 0.004
Sensitivity, n (%)	3 (12%)	4 (11%)	Chi sq, *p* = 0.86
Hypertensia/hypotensia, n (%)	7 (28%)	15 (39%)	Chi sq, *p* = 0.35
Insomnia, n (%)	20 (80%) *	19 (50%)	Chi sq, *p* = 0.02
Fatigue, n (%)	24 (96%) **	25 (66%)	Chi sq, *p* = 0.005
Attention deficit, n (%)	23 (92%) ***	19 (50%)	Chi sq, *p* = 0.0005
Memory deficit, n (%)	19 (76%)	22 (58%)	Chi sq, *p* = 0.14
Myalgia, n (%)	15 (60%)	14 (37%)	Chi sq, *p* = 0.07
Depression ^1^, n (%)	24 (96%) ***	13 (34%)	Chi sq, *p* = 0.000
Panic attacks, n (%)	5 (20%) *	1 (3%)	Chi sq, *p* = 0.03
Number of post-COVID symptoms	8.04 ± 2.23 ***	4.84 ± 3.50	F(1, 61) = 16.45, *p* = 0.000

Data are presented as mean ± SD. Significant differences between groups: * *p* < 0.05, ** *p* < 0.01, *** *p* < 0.001. ^1^ The “depression” parameter in the table corresponds to self-reported depression as reported by participants on the questionnaire, as opposed to depression diagnosed by a psychiatrist.

**Table 3:** The results of psychiatric testing.

Test	Parameter	Control	PCD	noPCD
**HDRS**	Hamilton score	4.0 ± 3.40	18.36 ± 3.66 *** ###	6.11 ± 3.52 *
**HADS**	Anxiety	4.42 ± 2.41	10.84 ± 3.25 *** ###	5.32 ± 3.59 ***
	Depression	3.47 ± 2.44	10.36 ± 4.78 *** ###	4.05 ± 2.89 ***
	Total score	7.89 ± 3.75	21.04 ± 7.40 *** ###	9.18 ± 4.73 ***

Data are presented as mean ± SD. Significant differences relative to the Control group: * *p* < 0.05, *** *p* < 0.001. Significant differences between the PCD and noPCD groups: ### *p* < 0.001.

**Table 4:** The factor structure in relation to the MPF percentage changes in post-COVID patients.

Factor	Eigenvalue	% Total Variance	Cumulative %	Brain Structures with Scores > 0.7
Factor 1	55.77	48.50	48.50	Anterior, Superior, and Posterior CR (L+R); Genu, Body, and Splenium of CC (L+R); Posterior thal. rad.(L+R); Tapetum (L+R); SLF (L+R); SFOF (L+R); Sagittal stratum (L+R); Anterior, Posterior, and Retrolenticular IC (L+R); FX stria terminalis (R); Superior, Middle, and Inferior Frontal WM (L+R); Lateral Fronto-Orbital (R); Superior Parietal WM (L+R); Middle and Inferior Occipital WM (L+R); Superior Occipital (L); Middle Temporal WM (L+R); Angular WM (L+R); Cingulum (cingulate) (L), Precentral (L+R); Pre-cuneus (L); Thalamus (L)
Factor 2	8.84	7.69	56.19	CST (L+R); Cerebral peduncle (L+R); Medial lemniscus (L+R); Pontine crossing tract (L+R); Inferior, Superior, and Middle CP (L+R); FX stria terminalis (L); Globus pallidus (L); Midbrain, Pons, Medulla
Factor 3	4.92	4.28	60.46	Middle Fronto-Orbital (R); Rectus (L+R)
Factor 4	4.20	3.65	64.12	Amygdala (L+R)
Factor 5	3.26	2.83	66.95	Cuneus (R)
Factor 6	2.85	2.48	69.42	Globus Pallidus (R); Putamen (R); SFOF (R); Anterior IC (L+R);
Factor 7	2.49	2.16	71.584	IFOF (L+R); Uncinate fasciculus (L+R)
Factor 8	2.22	1.93	73.52	Entorhinal area (L)
Factor 9	2.01	1.75	75.27	Caudate Nucleus (L+R)

**Table 5:** The parameters of multiple regressions predicting Hamilton scores in post-COVID patients.

Parameter	Total		PCD		noPCD	
Multiple R	0.64		0.70		0.36	
Multiple R^2^	0.41		0.50		0.127	
F	19.45		10.57		5.24	
*p*	0.0000		0.0000		0.0281	
**Variables in the model**	**β coefficient**	***p***	**β coefficient**	***p***	**β coefficient**	***p***
Number of acute symptoms			0.38	0.0019		
Number of post-COVID symptoms	0.56	0.0000			0.36	0.0281
Factor 7	−0.33	0.0010	−0.66	0.0003		

**Table 6:** The parameters of logistic regressions predicting the presence of PCD in post-COVID patients.

Statistic	Model 1		Model 2	
Likelihood ratio Chi sq. test, p	*p* = 0.0001		*p* = 0.00006	
Goodness of fit, Logit likelihood	−33.19		−30.32	
Goodness of fit, AIC	70.4		68.6	
Goodness of fit, BIC	76.8		77.2	
Odds ratio	6.77		14.03	
Correct predicted cases (%PCD/%noPCD)	60/78		68/87	
**Variables in the model**	**Wald stat., *p***	**Logit likelihood, Chi-sqr, *p***	**Wald stat., *p***	**Logit likelihood, Chi-sqr, *p***
Number of post-COVID symptoms	11.7, *p* = 0.0006	−34.9, 14.83, *p* = 0.0001	11.81, *p* = 0.0005	−34.9, 14.35, *p* = 0.0001
Factor 7	4.65, *p* = 0.03	−32.2, 5.4, *p* = 0.02	4.46, *p* = 0.03	−32.2, 5.41, *p* = 0.02
Gender	-	-	3.33, *p* = 0.05	−30.3, *p* = 0.047

## Data Availability

Data are unavailable due to privacy or ethical restrictions.

## Supplementary Materials

The following supporting information can be downloaded at: https://www.mdpi.com/article/10.3390/jcm13164692/s1, Questionnaire for Study Participation.
