# Supplementary material for: Demyelination in Patients with POST-COVID Depression
Source: J Clin Med. 2024 Aug 9;13(16):4692. doi: 10.3390/jcm13164692 (PMC11355865; doi:10.3390/jcm13164692)
Supplement: Supplementary file 1 [file jcm-13-04692-s001.zip › jcm-3081956-supplementary.pdf]

(English translation of original Russian version)

QUESTIONNAIRE FOR STUDY PARTICIPATION

**Last, first and middle names:**		**Sex:** ○ Male ○ Female
**Date of birth**: ________	**Date**: __________.	**Blood pressure** __________
**Height** _________	**Weight** __________	**Pulse** __________

	First time (if you were sick)	Second time (if you were sick)	Third time (if you were sick)
**Did a doctor tell you that you were sick (including asymptomatic) with a new coronavirus infection (COVID-19)?**	○ Yes ○ No	○ Yes ○ No	○ Yes ○ No
**COVID-19 status (check if appropriate):**			
• positive PCR test	☐	☐	☐
• IgG antibodies	☐	☐	☐
• IgM antibodies	☐	☐	☐
**When did you have COVID-19?** If you don't remember the exact dates, please indicate approximately, for example, early March 2021	Start date _______. End date _______.	Start date _______. End date _______.	Start date _______. End date _______.
**Have you ever had a coronavirus infection that affected your lungs? Have you been diagnosed with pneumonia?**	○ Yes ○ No	○ Yes ○ No	○ Yes ○ No
**Have you received treatment for coronavirus infection at home?**	○ Yes ○ No	○ Yes ○ No	○ Yes ○ No
**Have you been hospitalized with coronavirus infection?**	○ Yes ○ No	○ Yes ○ No	○ Yes ○ No
**Have you had mechanical ventilation?**	○ Yes ○ No	○ Yes ○ No	○ Yes ○ No
**Specify the manifestations of COVID-19 during illness (check as appropriate)**			
• asymptomatic	☐	☐	☐
• loss of smell	☐	☐	☐
• loss of taste	☐	☐	☐
• fever	☐	☐	☐
• difficulty breathing	☐	☐	☐

• cough	☐	☐	☐
• muscle weakness	☐	☐	☐
• muscle pain	☐	☐	☐
• headache	☐	☐	☐
• dizziness	☐	☐	☐
**What worries you after an infection? (check as appropriate)**	**First time** (if you were sick)	**If you were sick again**	**At this moment**
• new headache	☐	☐	☐
• dizziness	☐	☐	☐
• «brain fog»	☐	☐	☐
• loss of smell for more than 5 days	☐	☐	☐
• loss of taste for more than 5 days	☐	☐	☐
• loss of tactile sensitivity	☐	☐	☐
• increased blood pressure and heart rate	☐	☐	☐
• insomnia, sleep problems	☐	☐	☐
• increased fatigue	☐	☐	☐
• difficulty concentrating	☐	☐	☐
• memory impairment	☐	☐	☐
• joint and muscle pain	☐	☐	☐
• anxiety, depression	☐	☐	☐
• panic attacks	☐	☐	☐
**Other (specify)**	____________________________		
**Have you been vaccinated against coronavirus?**	○ Yes ○ No	○ Yes ○ No	○ Yes ○ No
**If yes, then when?** **(please mark the month and year)**	Date ________	Date ________	Date ________
**If "Yes", then which vaccine?**	Vaccine name ________	Vaccine name ________	Vaccine name ________
**Were you diagnosed with any of the following conditions before the COVID-19 pandemic? (check as appropriate)**			
Hypertension (stage)	☐		

Cerebrovascular diseases (stroke, chronic cerebral ischemia, stage)	☐
Traumatic brain injury (concussion, bruise, removal of hematoma)	☐
Encephalitis, meningitis	☐
Demyelinating disease (encephalomyelitis, multiple sclerosis)	☐
Other neurological disease	☐
Psychiatric illness	☐
Diabetes	☐
Oncological disease	☐
**If you noted anything, please specify the diagnosis and duration of the disease**	
**Other (specify)**	______________________________

Additional questions to describe the general condition of the body:

(you can only put a dash, if the answer is negative, if positive, then please describe in more detail)

Do you have hypersensitivity or contact allergies to odors? Do you have chronic smell disorders?	☐ Yes ☐ No<br>If yes, please briefly describe how this manifests itself:<br>____<br>____
Can you distinguish all colors?	☐ Yes ☐ No
Are you taking medications? Which? Is there a diagnosis due to which the doctor prescribed them?	☐ Yes ☐ No<br>If yes, please briefly list:<br>____<br>____
Do you have any chronic disease?	☐ Yes ☐ No<br>If yes, then please write the diagnosis, duration, stage (exacerbation or absence of symptoms at the moment):<br>____<br>____
If you have been diagnosed with cancer, are you currently undergoing treatment?	Please tell us briefly:<br>____<br>____
Have you recently experienced a stressful event (loss of a loved one, divorce, birth of a child (stress),	Please tell us briefly:<br>____<br>____

accident, fire, difficulties at work, dismissal)	
Have you visited a psychotherapist or psychologist? What was the goal of psychotherapy? Were any medications prescribed in connection with this request?	Please tell us briefly:<br>____<br>____
Do you have loss of appetite or sleep disturbances?	Please tell us briefly:<br>____<br>____
Have you noticed the predominance of any emotions (sadness, nervousness) or their pronounced frequent changes over the course of a week or month recently?	Please tell us briefly:<br>____<br>____
Do you visit an endocrinologist?	☐ Yes ☐ No<br>If yes, please briefly describe the reason for the complaint:<br>____<br>____

(Original Russian version)

АНКЕТА-ОПРОСНИК ДЛЯ УЧАСТИЯ В ИССЛЕДОВАНИИ

**Ф.И.О.**		**Пол**: ○ Мужской ○ Женский
**Дата рождения**: ________	**Дата анкетирования**: __________.	**Арт. давление** __________
**Рост**_________:	**Вес**__________	**Пульс** __________

	Первый раз (если болели)	Второй раз (если болели)	Третий раз (если болели)
**Говорил ли Вам врач, что Вы болели или перенесли, в том числе "на ногах", новую коронавирусную инфекцию (COVID-19)?**	○ Да<br>○ Нет	○ Да<br>○ Нет	○ Да<br>○ Нет
**COVID-19 статус (отметьте нужное):**			
• положительный ПЦР-тест	☐	☐	☐
• антитела IgG	☐	☐	☐
• антитела IgM	☐	☐	☐

**Когда Вы перенесли COVID-19?**<br><br>Если не помните даты точно, укажите приблизительно, например, начало марта 2021	Дата начала<br>________.<br><br>Дата окончания<br>________.	Дата начала<br>________.<br><br>Дата окончания<br>________.	Дата начала<br>________.<br><br>Дата окончания<br>________.
**Болели ли Вы коронавирусной инфекцией с поражением легких? Ставился ли диагноз «пневмония»?**	○ Да<br>○ Нет	○ Да<br>○ Нет	○ Да<br>○ Нет
**Получали ли Вы лечение коронавирусной инфекции дома?**	○ Да<br>○ Нет	○ Да<br>○ Нет	○ Да<br>○ Нет
**Были ли Вы госпитализированы по поводу коронавирусной инфекции?**	○ Да<br>○ Нет	○ Да<br>○ Нет	○ Да<br>○ Нет
**Проводилась ли ИВЛ?**	○ Да<br>○ Нет	○ Да<br>○ Нет	○ Да<br>○ Нет
**Уточните проявления COVID-19 во время заболевания (отметьте нужное)**			
• бессимптомно	☐	☐	☐
• потеря обоняния	☐	☐	☐
• потеря вкуса	☐	☐	☐
• температура	☐	☐	☐
• трудности дыхания	☐	☐	☐
• кашель	☐	☐	☐
• мышечная слабость	☐	☐	☐
• мышечные боли	☐	☐	☐
• головная боль	☐	☐	☐
• головокружение	☐	☐	☐

**Что беспокоит вас после перенесённой инфекции?(отметьте нужное)**	**Первый раз**<br><br>**(если болели)**	**Если болели**<br><br>**повторно**	**На текущий момент**
• впервые возникшая головная боль	☐	☐	☐
• головокружение	☐	☐	☐
• «мозговой туман»	☐	☐	☐
• нарушение обоняния более 5 дней	☐	☐	☐
• нарушение вкуса более 5 дней	☐	☐	☐
• нарушение тактильной чувствительности	☐	☐	☐
• повышения давления, сердцебиения	☐	☐	☐
• бессонница, нарушения сна	☐	☐	☐
• повышенная утомляемость	☐	☐	☐
• трудности с концентрацией внимания	☐	☐	☐
• нарушения памяти	☐	☐	☐
• суставные или мышечные боли	☐	☐	☐
• беспокойство, депрессия, тревога	☐	☐	☐
• панические атаки	☐	☐	☐
**Другое (уточните)**	______________________________		
**Вакцинировались ли Вы от коронавируса?**	○ Да<br>○ Нет	○ Да<br>○ Нет	○ Да<br>○ Нет
**Если «Да», то когда?**<br><br>**(отметьте месяц и год)**	Дата ________.	Дата ________.	Дата начала ________.
**Если «Да», то какой вакциной?**	Название вакц. ________.	Название вакц ________.	Название вакц ________.

**Было ли у Вас диагностировано какое-либо из нижеперечисленных заболеваний до пандемии COVID-19? (отметьте нужное)**	
Гипертоническая болезнь (стадия)	☐
Цереброваскулярные заболевания (инсульт, хроническая ишемия мозга, стадия)	☐
Черепно-мозговая травма (сотрясение, ушиб, удаление гематомы)	☐
Энцефалит, менингит	☐
Демиелинизирующее заболевание (энцефаломиелит, рассеянный склероз)	☐
Другое неврологическое заболевание	☐
Психиатрическое заболевание	☐
Сахарный диабет	☐
Онкологическое заболевание	☐
**Если отметили что-либо, конкретизируйте диагноз и длительность заболевания**	
**Другое (уточните)**	________________________________

**Дополнительные вопросы по описанию общего состоянии организма:**<br>***(можно поставить только прочерк, если ответ отрицательный, если положительный, то опишите, пожалуйста, подробнее)***	
Есть ли у Вас повышенная чувствительность или контактная аллергия на запахи? Есть ли у Вас хронические нарушения обоняния?	☐ Да ☐ Нет<br>Если да, то, пожалуйста, кратко опишите, как это проявляется:<br>____<br>____
Можете ли Вы различать все цвета?	☐ Да ☐ Нет
Принимаете ли Вы препараты? Какие? Есть ли диагноз, благодаря которому их назначил врач?	☐ Да ☐ Нет<br>Если да, то, пожалуйста, кратко перечислите:

	____________________<br>____________________
Есть ли у Вас любое хроническое заболевание?	☐ Да ☐ Нет<br>Если да, то, пожалуйста, напишите диагноз, длительность, стадию (обострение или отсутствие симптомов на данный момент):<br>____________________<br>____________________
Если у Вас было диагностировано онкологическое заболевание, то проходите ли Вы лечение сейчас?	Пожалуйста, кратко расскажите:<br>____________________<br>____________________
Перенесли ли Вы недавно стрессовое событие (утрата близкого, развод, рождение ребенка (нагрузка), авария, пожар, сложности на работе, увольнение)	Пожалуйста, кратко расскажите:<br>____________________<br>____________________
Посещали ли Вы психотерапевта или психолога? Какой была цель психотерапии? Были ли назначены препараты в связи с данным обращением?	Пожалуйста, кратко расскажите:<br>____________________<br>____________________
Есть ли у Вас нарушение аппетита или сна?	Пожалуйста, кратко расскажите:<br>____________________<br>____________________
Замечаете ли Вы преобладание каких-либо эмоций (грусть, нервозность) или их выраженную частую перемену в течении недели или месяца в последнее время?	Пожалуйста, кратко расскажите:<br>____________________<br>____________________

Посещаете ли Вы эндокринолога?	☐ Да ☐ Нет<br>Если да, то, пожалуйста, кратко опишите причину жалоб:<br>______________________________<br>______________________________
